# Role of Cln1 during melanization of *Cryptococcus neoformans*

**DOI:** 10.3389/fmicb.2015.00798

**Published:** 2015-08-12

**Authors:** Rocío García-Rodas, Nuria Trevijano-Contador, Elvira Román, Guilhem Janbon, Frédérique Moyrand, Jesús Pla, Arturo Casadevall, Oscar Zaragoza

**Affiliations:** ^1^Mycology Reference Laboratory, National Centre for Microbiology, Instituto de Salud Carlos IIIMajadahonda, Spain; ^2^Department of Microbiology, Pharmacy Faculty, Complutense University of MadridMadrid, Spain; ^3^Unité Biologie et Pathogénicité Fongiques, Institut PasteurParis, France; ^4^Department of Molecular Microbiology and Immunology, Johns Hopkins Bloomberg School of Public Health, BaltimoreMD, USA

**Keywords:** melanin, cell wall, laccase, *Cryptococcus CLN1*

## Abstract

*Cryptococcus neoformans* is an opportunistic fungal pathogen that has several well-described virulence determinants. A polysaccharide capsule and the ability to produce melanin are among the most important. Melanization occurs both *in vitro*, in the presence of catecholamine and indole compounds, and *in vivo* during the infection. Despite the importance of melanin production for cryptococcal virulence, the component and mechanisms involved in its synthesis have not been fully elucidated. In this work, we describe the role of a G1/S cyclin (Cln1) in the melanization process. Cln1 has evolved specifically with proteins present only in other basidiomycetes. We found that Cln1 is required for the cell wall stability and production of melanin in *C. neoformans*. Absence of melanization correlated with a defect in the expression of the *LAC1* gene. The relation between cell cycle elements and melanization was confirmed by the effect of drugs that cause cell cycle arrest at a specific phase, such as rapamycin. The *cln1* mutant was consistently more susceptible to oxidative damage in a medium that induces melanization. Our results strongly suggest a novel and hitherto unrecognized role for *C. neoformans* Cln1 in the expression of virulence traits.

## Introduction

Melanins are dark, hydrophobic, negatively charged pigments that are widespread in nature (Nosanchuk and Casadevall, 2003). The structure of melanins remain unidentified, so they are defined as pigments that are complex polymers with a high molecular mass, amorphous nature, acid resistance, and with a stable free radical signal (White, 1958; Jacobson, 2000; Wakamatsu and Ito, 2002). There are several types of melanins: eumelanins, pheomelanins, alomelanins, and piomelanins (Carreira et al., 2001; Wakamatsu and Ito, 2002; Plonka and Grabacka, 2006). Some fungal melanins derive from the precursor molecule 1,8-dihydroxynaphthalene (DHN), such as those from *Wangiella dermatitidis* and *Alternaria alternata*, and are produced from endogenous substrates. Alternatetively, some fungi produce melanin from L-3,4 dihydroxyphenylalanine (L-DOPA; Eisenman and Casadevall, 2012).

Melanins contribute to the virulence of pathogens and increase resistance to environmental damage as well (Rosa et al., 2010). In *Cryptococcus neoformans*, genes involved in melanization contribute to host death and dissemination from lungs (Salas et al., 1996; Noverr et al., 2004). Melanization in *C. neoformans* is catalyzed by laccase, a cell wall associated diphenoloxidase that catalyzes the oxidation of diphenolic compounds to their respective quinones (Chaskes and Tyndall, 1978). However, *C. neoformans* cannot use tyrosine as a substrate to produce melanin (Nurudeen and Ahearn, 1979). Two laccase encoding genes, *LAC1* and *LAC2*, have been identified in *C. neoformans*, with *LAC1* being the main producer of melanin (Pukkila-Worley et al., 2005). In addition, other genes including *VPH1, CCC2, ATX1, CHS3, MBF1*, and *XRN1* are also required for melanization, although in most cases their mode of action is not well characterized (Erickson et al., 2001; Zhu et al., 2003; Walton et al., 2005; Wollschlaeger et al., 2014).

We have been recently interested in the role of cell cycle elements in the regulation of the expression of cryptococcal virulence factors, with special emphasis on the capsule. During the course of the characterization of *cln1*, a cyclin mutant of *C. neoformans* (Garcia-Rodas et al., 2014) we found out that it was unable to melanize. Cln1 is a G1 cyclin in *C. neoformans*. While it is not essential for cell cycle progression, it is responsible for a delay in the transition between G1 and S phases (Garcia-Rodas et al., 2014). Little is known about cell cycle progression in *C. neoformans*, but during exponential phase, budding and DNA synthesis occur simultaneously (Berman, 2006). However, at the end of exponential phase, budding is delayed and cells are arrested at G1 or G2 phases (Takeo et al., 1995; Virtudazo et al., 2010). We observed that capsule enlargement is coordinated with cell cycle progression. Therefore, and given that *cln1* was unable to melanize, we decided to study the relationship between this particular cyclin of *C. neoformans* and the ability of the yeast to produce melanin. Our results demonstrate that the production of melanin seems to be regulated through a complex pathway in which cell wall stability is essential. In addition, drugs that cause cell cycle arrest in G1/S phase results in defects in melanization.

## Materials and Methods

### Strains and Culture Conditions

*Cryptococcus neoformans* var. *grubii* H99 strain (Perfect et al., 1980) and the mutant CNAG_06092 (*cln1*) obtained from a collection of mutants deposited at the ATCC by Dr. Madhani (Liu et al., 2008) were used in this study. In addition, the reconstituted strain, *cln1::CLN1* was generated by biolistic transformation as described in (Garcia-Rodas et al., 2014).

The strains were routinely grown in liquid Sabouraud medium (Oxoid LTD, UK) at 30°C or 37°C with moderate shaking (150 rpm). To induce melanization, strains were grown protected from light in chemically defined minimal medium (15 mM dextrose, 10 mM MgSO_4_, 29.4 mM KHPO_4_, 13 mM glycine, and 3 μM thiamine, pH 5.5) with 1 mM L-DOPA (Sigma–Aldrich, St. Louis, MO, USA) and incubated at 30°C with shaking. In some cases, the same medium without L-DOPA was used as a control.

For solid media, 1.5% agar was added to the medium. Yeast suspensions were prepared at 2 × 10^7^/mL in PBS. Serial 1:10 dilutions were performed and 5 μL from each dilution were spotted. Plates were incubated at 30 and 37°C and pictures were taken daily. In some cases, 1M Sorbitol or Congo Red (0.05; 5 and 10 mg/mL) were added to Sabouraud agar plates.

### Phylogenetic Tree

We performed evolutionary studies of the Cln1 protein sequence using PhylomeDB (http://phylomedb.org; Huerta-Cepas et al., 2008, 2011) by Dr. Gabaldón’s lab (Comparative Genomics Group at Centre for Genomic Regulation, Barcelona, Spain). PhylomeDB is an algorithm that allows the visualization of the evolution of a specific gene by comparison of multiple genomes. After introduction of the protein sequence, the software performs multiple alignments and creates a tree where the evolution of a specific protein (duplication and speciation events) can be visualized and paralogs and orthologs identified.

### Wheat Germ Agglutinin Staining

The presence of chitin-like structures, WGA staining was ascertained as described in (Rodrigues et al., 2008a). Briefly, *C. neoformans* cells with enlarged capsule (incubated in 10% Sabouraud in 50 mM MOPS buffer pH 7.3 overnight at 30°C; Zaragoza and Casadevall, 2004) were washed in PBS and suspended in 4% *p*-formaldehyde for 30 min at room temperature. The fixed cells were washed and incubated in 100 μL of PBS containing 5 μg/mL of WGA conjugated to Alexa-594 (Molecular Probes, Invitrogen) for 1 h at 37°C. Next, cells were again washed and suspended in 100 μL of PBS. Cell suspensions were mounted over glass slides and photographed with a SP5 confocal microscope (Leica Microsystems).

### Melanin Production

Melanin production was assessed on both liquid and solid chemically defined minimal medium (see above) supplemented with L-DOPA. After 24–48 h, melanization was assessed by acquisition of a dark color of the cultures, compared to parallel cultures without L-DOPA. For melanin production on solid medium, yeast suspensions were prepared at 2 × 10^7^/mL in PBS. Serial 1:10 dilutions were performed and 5 μL from each one were spotted on L-DOPA plates and on Sabouraud dextrose agar. Plates and cultures were incubated at 30 and 37°C protected from light. Pictures of either the plates or liquid cultures were taken daily.

### Laccase Activity Assay

*Cryptococcus neoformans* strains were incubated in 15 mL of chemically defined minimal medium with L-DOPA overnight at 30°C with shaking (150 rpm) and protected from light. Laccase activity was assessed as described in (Alvarado-Ramirez et al., 2008) with some modifications. Pelleted cells were suspended in 2 mL of PBS and divided in two equal fractions; one was used as a negative control by inactivating the laccase activity with 5% β-mercaptoethanol for 2 h at 37°C. Then, both aliquots were disrupted using a Fast Prep homogenizer (MP Biomedicals) 0.5 grams of 425–600 μm Ø glass beads (Sigma–Aldrich, St. Louis, MO, USA). A minimum of six cycles of 45 s were performed (with intervals of 4 min in ice). The mixture was centrifuged at 13,800 *g* for 10 min at 4°C. Supernatants were conserved at 4°C until enzymatic determination was performed. To quantify the laccase activity, 100 μL of every sample were placed in a 96 well-plate (Costar, NY, USA), and 7 μl of a 20 mM L-DOPA solution were added to each well. The microplate was incubated at 25°C with moderate shaking in an iEMS Spectrophotometer (Thermofisher) for 18 h. Optical density at 450 nm was measured every 15 min. Protein concentration of the extracts was determined with the Bradford method using the Quick Start Bradford Protein Assay (BioRad, CA, USA). Specific activity was expressed as mUAbs/min/μg protein.

### Quantification of the Expression of the *LAC1* Gene by Real-Time PCR

Yeast cells were grown for 3 days in minimal medium at 30°C with shaking (150 rpm) as described above. RNA extraction was performed using Trizol reagent protocol (Ambion RNA by life technologies) with some modifications. The cells were disrupted with glass beads using a FastPrep-24 (MP^TM^) for 5 min, alternating 20 s shaking with 1 min on ice. The RNAs were quantified and qualified using the Nanodrop 8000 Spectrophotometer (Thermo scientific). cDNAs were generated using the iScrip^TM^ cDNA synthesis Kit (Bio-Rad) following the manufacturer’s recommendations. The RT-PCR was performed using SsoAdvanced^TM^ Universal SYBR^R^ Green Supermix (Bio-Rad) using the *LAC1* specific primers (LAC1F AGAAGGGAAGGAAGGTGATG and LAC1R1 TATACCTCACAACCGCCAAT described in (Alanio et al., 2011) in a total volume of 20 μl, in a Light Cycler^R^ 480 (Wang et al., 2006). As control, 18s specific primers were used (18sF CCGTTGCTAGAGGTGAAATTCTTAG and 18sR ATCTAATCGTTTTTGATCCCCTAAC). The RT-PCR conditions were 95°C for 10 min and 40 cycles of amplification (95°C for 15 s, 58°C for 1 min). Differences in gene expression were calculated using the 2^-ΔΔCt^ method.

### Susceptibility to Oxidative Stress

*Cryptococcus neoformans* strains were incubated in L-DOPA liquid medium for 48 h at 30°C with shaking (150 rpm). Suspensions were prepared at 2 × 10^3^ cells/mL in PBS. Hydrogen peroxide (Fluka, St. Louis, MO, USA) was added at different final concentrations (0.5, 1, and 2 mM) and cells were incubated at 30°C for 1 h. Samples without hydrogen peroxide were incubated in parallel as controls. Each sample was carried out in triplicate. Then, 50 and 100 μL were plated on Sabouraud agar plates and incubated at 30°C for 2 days. The number of colony forming units (CFU) was enumerated, and viability was calculated as the percentage of colonies obtained in the treated samples compared with the untreated controls.

### Melanin Production in the Presence of Cell Cycle Inhibitors

*Cryptococcus neoformans* strain H99 cells were grown in 5 mL of Sabouraud at 30°C with agitation. Cells were washed and transferred to L-DOPA liquid medium containing different rapamycin concentrations (0.5 and 1 μg/mL, Sigma–Aldrich, St. Louis, MO, USA), which induces G1 arrest. The same amount of DMSO (Sigma–Aldrich, St. Louis, MO, USA) was added to the control cultures (without the drug) since rapamycin is diluted in this solvent. Cells were grown overnight at 30°C and pictures were taken daily to asses melanization.

To assess cell viability after treatment with the different concentrations of rapamycin cells were stained with propidium iodide at a final concentration of 5 μg/ml and quantified by flow cytometry using a FacsCalibur flow cytometer (BD, Biosciences, Worburn, MA, USA). Data were processed using CellQuest (BD, Biosciences) and FlowJo (Tree Star Inc, Ashland, OR, USA) softwares.

### Growth Curves in L-DOPA Liquid Medium in the Presence of Cell Cycle Inhibitors

Suspensions of *C. neoformans* strain H99 at 5 × 10^5^/mL were prepared in chemically defined minimal medium with 1 mM L-DOPA containing different concentrations of rapamycin (0, 0.5, or 1 μg/mL). Control suspensions of *C. neoformans* cells in chemically defined medium with DMSO were carried out in parallel in each case. Cells were grown for 48 h at 30°C and optical density at 540 nm was assessed every hour using an iEMS Spectophotomer (Thermofisher). Graphs were plotted using Graph Pad Prism 5.

### Statistical Analysis

Scatter plot graphs of cell sizes were done using Graph Pad Prism 5 (La Jolla, CA, USA), and statistical differences were assessed with *t*-test. A *p*-value <0.05 was considered significant.

## Results

### Phylogenetic Analysis of Cln1

**FIGURE 1:**
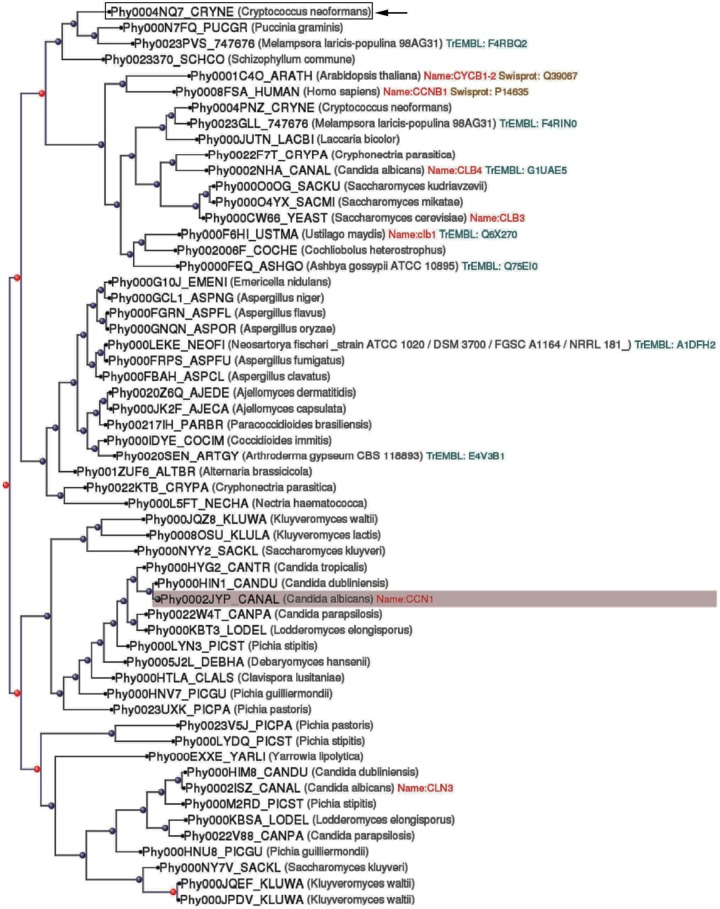
**Phylogenetic tree of G1/S cyclin (Cln1).** A phylogenetic tree analysis of *Cryptococcus neoformans* Cln1 protein sequence was performed using PhylomeDB as described in Materials and Methods. The sequence of *Candida albicans* Ccn1 was used as root. The location of the *C. neoformans* Cln1 sequence is highlighted with a black arrow. Red dots indicate duplication events, and in consequence, appearance of paralogs, and blue dots denote speciation events and appearance of orthologs.

Previous work from our laboratory had focus on the role of Cln1 on capsule production (Garcia-Rodas et al., 2014). During the characterization of the role of this protein on *C. neoformans* capsule formation, we found that the corresponding mutant strain presented some abnormal phenotypes that were not expected to be related with cell cycle regulation, such as a defect in melanization. In consequence, we decided to further characterize phenotypically this mutant. First, we performed a blast comparison using PhylomeDB, an algorithm that allows the generation of phylogenetic evolutionary trees and the identification of gene paralogs and orthologs. The results showed that although Cln1 had evolutionary similarities with cyclins from other organisms, in *C. neoformans*, this cyclin had evolved specifically with proteins present only in other basidiomycetes after two duplication events from a common ancestor (**Figure 1**). Therefore, it is possible that this protein has new roles in *C. neoformans* development and/or pathogenesis independently of cell cycle functions.

### Growth and Morphological Characteristics of *cln1* Mutant

**FIGURE 2:**
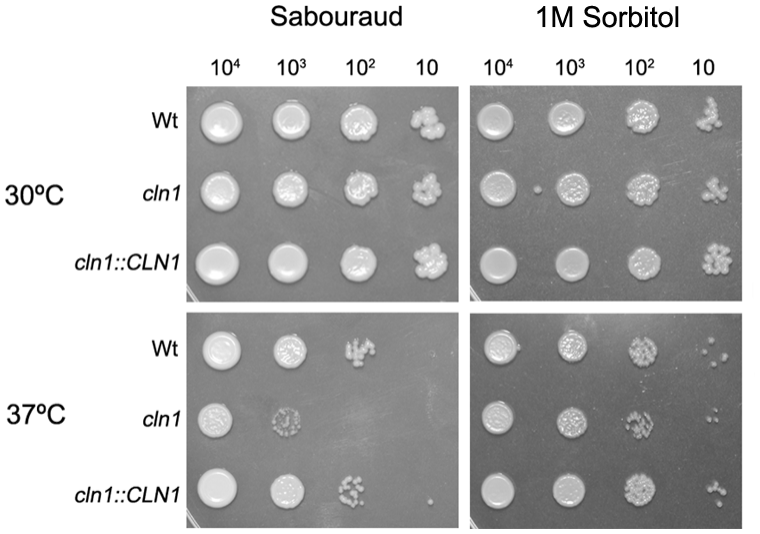
**Growth of *cln1* mutant on solid medium.** Panels show fungal growth on Sabouraud agar plates, Sabouraud agar plates supplemented with 1 M Sorbitol, at 30°C (upper panels) and at 37°C (lower panels). Upper numbers indicate the number of cells placed in each spot. Pictures were taken after 48 h.

**FIGURE 3:**
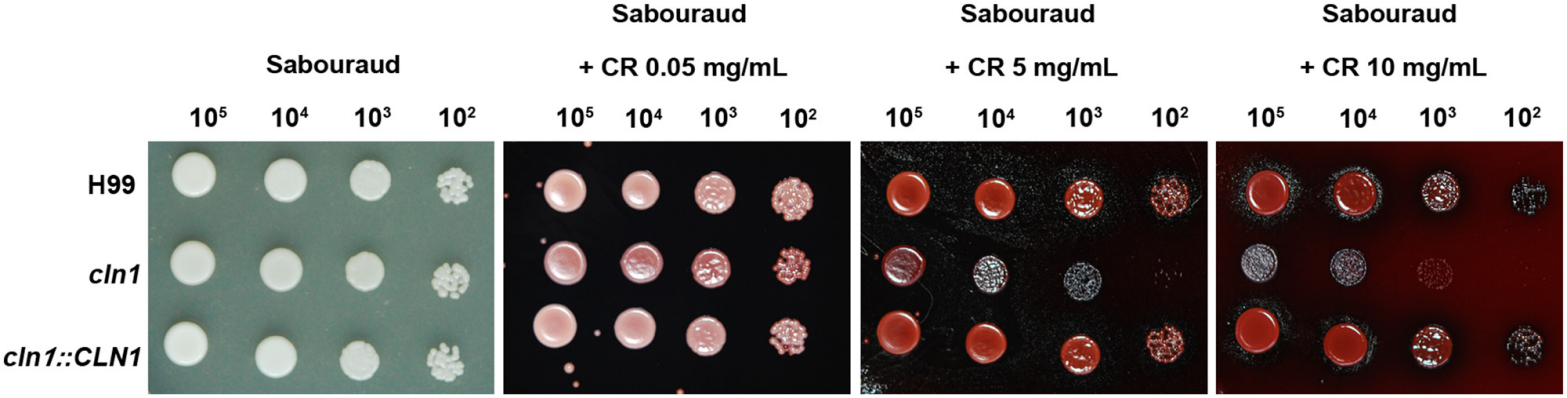
**Cell wall stability assay.** Growth of wt, *cln1* and the reconstituted strains on Sabouraud agar plates with different Congo Red (CR) concentrations. Plates were incubated at 30°C and pictures were taken after 48 h.

We investigated the growth rate of *cln1* at different temperatures. The mutant *cln1* showed growth defects at 37°C in Sabouraud (**Figure 2**). However, this defect was partially restored when 1 M Sorbitol was added (**Figure 2**). Interestingly, sorbitol did not restore other morphological defects described for this mutant, such as an engrossment of the bud neck or enlarged cell size (data not shown). This result suggested that *cln1* had cell wall defects. Therefore, we investigated if *cln1* was more susceptible to agents that affect the stability of the cell wall, such as Congo Red (CR). As shown in **Figure 3**, CR inhibited *cln1* growth in a dose dependent manner, which was linked to possible cell wall alterations. We tested other cell wall disturbing compounds, such as tunicamycin, or osmotic stress (NaCl), but we did not find any difference in the growth between any of the strains.

**FIGURE 4:**
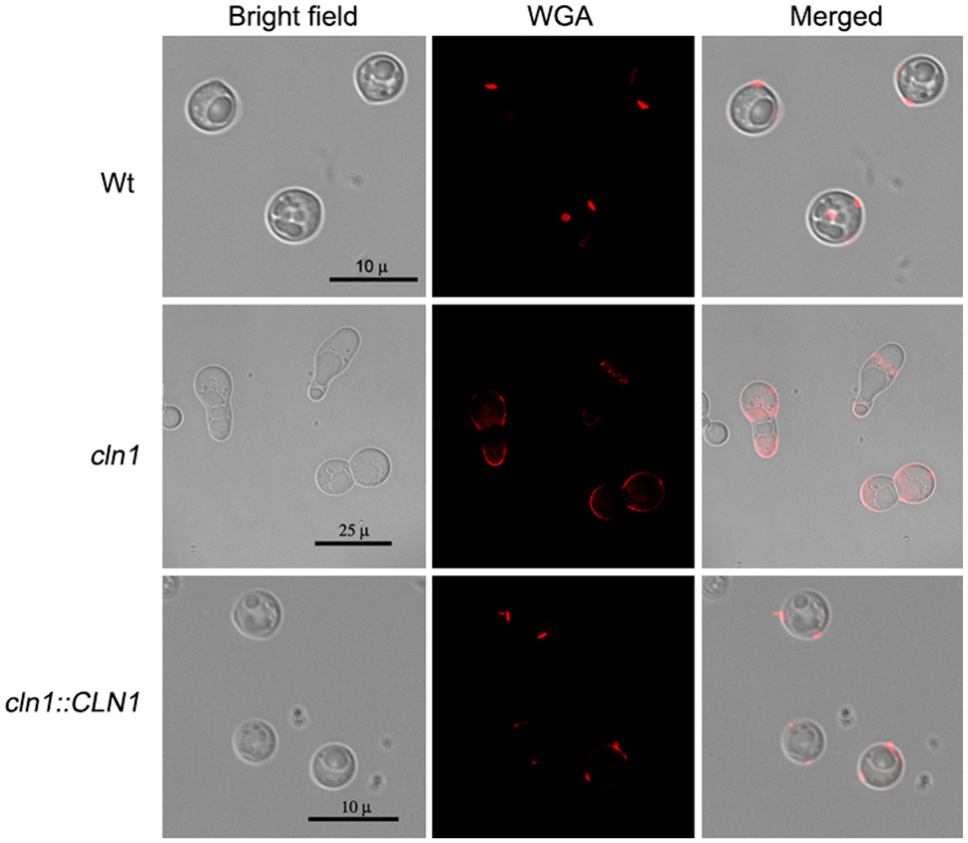
**Binding of wheat germ agglutinin to *C. neoformans* strains.** Panels show representative cells of each strain grown in capsule inducing medium at 30°C for 24 h stained with WGA as described in Section “Materials and Methods.”

Chitin-like molecules in fungi are polymerized by chitin synthases, which use cytoplasmic pools of UDP-GlcNAc (*N*-acetylglucosamine) to form β-1,4-linked oligosaccharides and large polymers. In *C. neoformans*, the final cellular site of chitin accumulation is the cell wall, although some protuberances of this structure have been also found in the capsule (Rodrigues et al., 2008a). The chitin-like structures in the capsule can be visualized by the binding of fluorescent wheat germ agglutinin, which binds to sialic acids and β-1,4-*N*-acetylglucosamine (GlcNAc) oligomers (Rodrigues et al., 2008a). We used WGA to ascertain whether these structures were also present in the *cln1* mutant. Cells from the wild type and *cln1::CLN1* strains bound WGA at the neck between the mother cell and the bud. In contrast, in cells from the *cln1* strain, these structures were delocalized and were found all over the cell giving also a more intense fluorescent signal (**Figure 4**).

### Melanin Production and Laccase Activity

**FIGURE 5:**
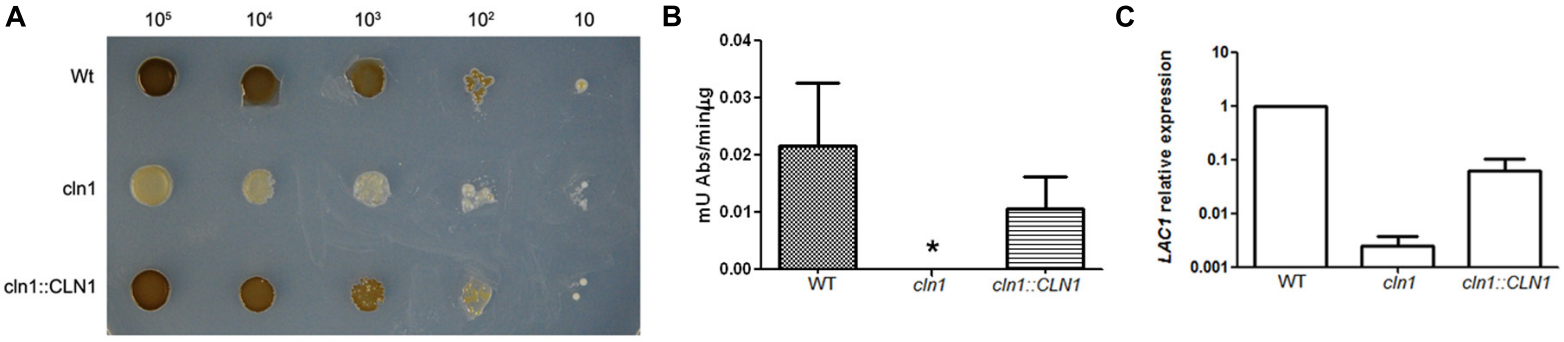
**Melanin production and laccase activity. (A)** Growth of *C. neoformans* strains on L-3,4 dihydroxyphenylalanine (L-DOPA) agar plates at 30°C. **(B)** Laccase activity of *C. neoformans* strains. A *p*-value <0.05 is indicated with an asterisk. **(C)** *LAC1* gene expression. Cells from WT, *cln1* and *CLN::CLN1* strains were grown in minimal media for 3 days at 30°C and RNA samples were isolated. cDNAs were obtained and relative expression of the *LAC1* gene was measured by real time PCR.

During the phenotypic characterization of *cln1*, we observed that Cln1 was required for the accumulation of melanin (**Figure 5A**). Wild type and *cln1::CLN1* strains produced melanin after 3–4 days of incubation at 30°C on L-DOPA agar plates (**Figure 5A**) while *cln1* mutant was unable to accumulate melanin. In liquid medium, we found that melanization of the wt and *cln1::CLN1* strains was visible after 24 h of incubation with L-DOPA, while the *cln1* mutant did not accumulate melanin.

To get insights about this phenotype, we measured the laccase activity in the wild type and mutant strains (Zhu et al., 2001). In agreement with the visual observation showing absent melanization, the *cln1* mutant had no laccase activity in the conditions tested. This defect was recovered in the reconstituted strain (**Figure 5B**).

We then investigated if the defect in laccase activity was due to a defect at the transcriptional level. We measured the relative levels of the mRNA of the *LAC1* gene by real-time PCR. For this purpose, total RNA was isolated from cells grown in minimal medium and cDNA was obtained using standard protocols. We then used this cDNA as template to quantify the levels of the *LAC1* mRNA. The expression of the *LAC1* gene in the *cln1* mutant was significantly lower than in the WT strain, and this defect was partially complemented in the reconstituted strain (**Figure 5C**), indicating that absence of melanization was mainly due to a defect in the transcription of the *LAC1* gene.

### Melanin Protects *C. neoformans* from Oxidative Damage

**FIGURE 6:**
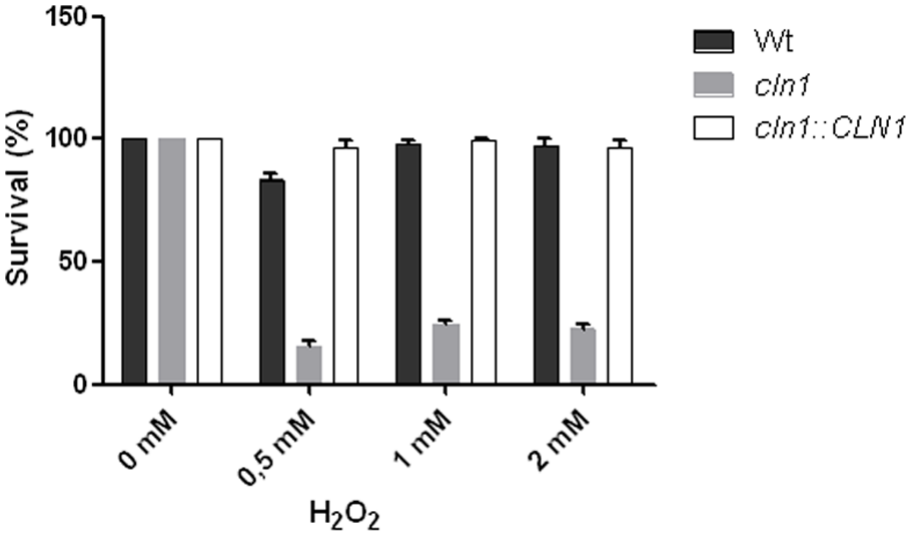
**Susceptibility to oxidative stress.** Bars show different susceptibility of *C. neoformans* strains grown in L–DOPA liquid medium and incubated in the presence of different H_2_O_2_ concentrations for 1 h.

Laccase and melanin confer resistance to oxidative damage (Wang et al., 1995), and consequently we tested the susceptibility of Cln1 sufficient and deficient strains to H_2_O_2_. Cells from WT and *cln1::CLN1* grown for 48 h in L-DOPA liquid medium showed resistance to oxidative stress at all concentrations tested (0.5, 1, and 2 mM, **Figure 6**). In contrast, *cln1* cells grown in the same conditions showed a five-fold reduction in survival when exposed to H_2_O_2_, even when using low H_2_O_2_ concentrations such as 0.5 mM (**Figure 6**). In contrast, *cln1* and WT cells grown in Sabouraud liquid medium showed no statistical significant differences in survival when incubated with different concentrations of H_2_O_2_ (data not shown).

### Melanin Production in the Presence of Cell Cycle Inhibitors

**FIGURE 7:**
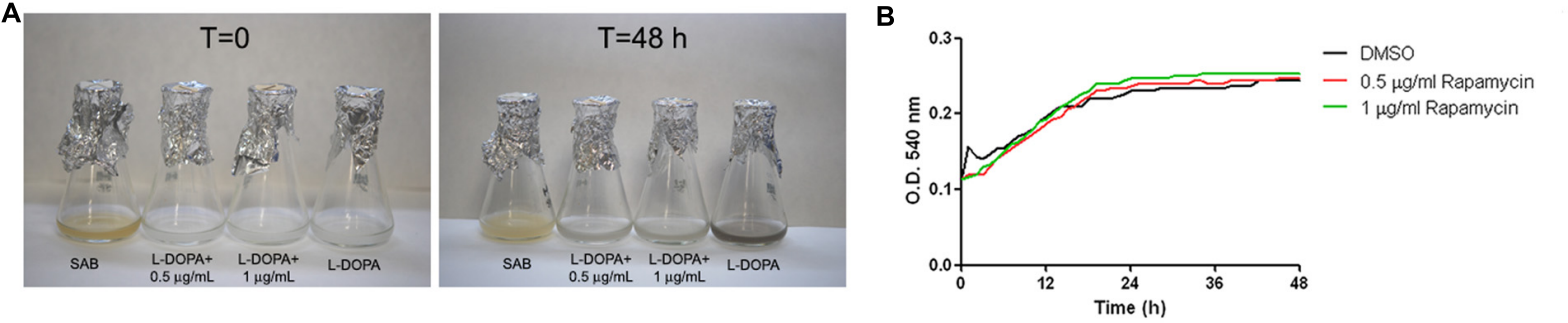
**Effect of rapamycin on melanization and growth of *C. neoformans*. (A)** Panels show cultures of *C. neoformans* in L-DOPA liquid medium containing different rapamycin concentrations at 0 and 48 h. Cultures with DMSO were carried out in parallel since rapamycin was dissolved in DMSO. **(B)** Growth of *C. neoformans* in L-DOPA medium containing different rapamycin concentrations. Optical density (OD) was measured at 540 nm every hour for 48 h. Black line, growth in control medium (minimal medium + DMSO); red line, rapamycin 0.5 μg/mL; green line, rapamycin 1 μg/mL.

The lack of melanization in *cln1* suggested a link between cell cycle elements and the capability to produce and/or accumulate melanin. Therefore we examined if melanin production was affected by drugs that cause cell cycle arrest. First, we tested melanization in L-DOPA liquid cultures containing different concentrations of rapamycin (0.5 and 1 μg/mL), which is an inhibitor of the TOR signaling pathway and causes G1 arrest. As shown in **Figure 7**, rapamycin inhibited melanin production by *C. neoformans* in L-DOPA liquid cultures (**Figure 7A**). To assess if lack of melanization was due to an inhibitory effect of rapamycin on growth rate, we studied growth in L-DOPA liquid medium with different concentrations of rapamycin and observed that this drug had no inhibitory effect on the growth rate of *C. neoformans* in L-DOPA liquid medium (**Figure 7B**).

## Discussion

During the phenotypic characterization of the *cln1* mutant, we found that it was unable to synthesize melanin in the presence of substrates such as L-DOPA. The production of melanin is known to confer protection against different stress conditions (Jacobson and Tinnell, 1993; Emery et al., 1994; Wang and Casadevall, 1994a,b; Rosas and Casadevall, 1997). Some factors that regulate melanization have already been described (Alspaugh et al., 1997, 1998; D’Souza et al., 2001; Nosanchuk et al., 2001; Vidotto et al., 2002; Tangen et al., 2007; Mauch et al., 2013). In *C. neoformans* this process only takes place in the presence of specific substrates, mainly diphenolic compounds (Chaskes and Tyndall, 1975, 1978). Melanization depends on the expression of a gene that encodes a diphenol oxidase, called laccase, and the expression of this gene is repressed by glucose (Nurudeen and Ahearn, 1979). Our initial finding suggested that melanization is in fact under the regulation of a more complex circuit, and for that reason, we decided to characterize this phenotype in detail.

Consistent with its role in cell cycle regulation, the cyclin mutant displayed growth defects, especially at 37°C. This might be explained by the fact that some genes that are expressed at the G1/S phase boundary are involved in cell wall biosynthesis (Igual et al., 1996). This idea is supported by the restoration of the growth defect phenotype after the addition of osmotic stabilizers (sorbitol) to the media and the higher susceptibility to agents that alter the stability of the cell wall such as CR. We tested if the *cln1* mutant had any defect in the activation of MAPK that are involved in the response to cell wall alterations (such as Mkc1 and Cek1; Navarro-Garcia et al., 1995, 2005; Eisman et al., 2006; Roman et al., 2009), but our preliminary data showed no difference in the activation of these kinases between any of the strains tested in this work (data not shown).

Laccase in *C. neoformans* is synthesized in the cytoplasm (Jacobson, 2000; Garcia-Rivera et al., 2005; Eisenman et al., 2007) and then it is believed to be transported in vesicles to the cell wall, where it is finally tightly linked through disulfide or thioester bonds. (Zhu et al., 2001; Waterman et al., 2007; Rodrigues et al., 2008b). The mutant *cln1* presents cell wall defects and miss localization of chitin like structures as evidenced by the delocalized binding of WGA. Therefore, we believe that laccase is not correctly linked to cell wall and thus, the *cln1* mutant strain is unable to produce melanin. This phenotype was also confirmed independently by testing the susceptibility of *cln1* to stress factors, such as H_2_O_2_, and the effect of cell cycle inhibitors on melanization. Our results are in agreement with previous findings that described that melanization depends on proteins involved in cell wall structure, such as Chs3 (Walton et al., 2005).

Curiously, we observed that *cln1* could grow normally at 37°C in minimal medium. Growth curves performed with *C. neoformans* WT strain in the presence of cell cycle inhibitors in minimal medium revealed that rapamycin had no effect on growth rate. These results suggest that neither rapamycin, nor Cln1 regulate growth rate in minimal medium, although they are necessary to melanize. Cln1 has been involved in the regulation of cell cycle in *C. neoformans* (Virtudazo et al., 2010, 2011; Garcia-Rodas et al., 2014). However, our findings suggest that in certain conditions, cell cycle progression does not depend on Cln1 nor Tor proteins, and these proteins seem to have other functions in other metabolic processes, such as melanin production, independently of cell cycle regulation.

Finally, our results show a plausible link between cell cycle and melanin production. The phylogenetic analysis showed that Cln1 had evolved differently compared to cyclins of other microorganisms, and that this evolutionary pattern is only present in basidiomycetes. This result suggests that Cln1 of *C. neoformans* has acquired specific functions that are not present in other microorganisms, and thus, it could regulate processes, such as melanin production, characteristic of *C. neoformans*. Cln1 modulates G1/S transition in *C. neoformans* and its absence leads to defects on virulence determinants, such as melanin production. This study offers new directions in cryptococcal virulence research and the possibility of new drug targets involved in cell cycle progression.

## Conflict of Interest Statement

The authors declare that the research was conducted in the absence of any commercial or financial relationships that could be construed as a potential conflict of interest.

## References

[B1] Alanio A. Desnos-Ollivier M. Dromer F. (2011). Dynamics of *Cryptococcus neoformans*-macrophage interactions reveal that fungal background influences outcome during cryptococcal meningoencephalitis in humans. *MBio* 2:e00158 10.1128/mBio.00158-11 PMC3149853 21828220

[B2] Alspaugh J. A. Perfect J. R. Heitman J. (1997). *Cryptococcus neoformans* mating and virulence are regulated by the G-protein alpha subunit GPA1 and cAMP. *Genes Dev.* 11 3206–3217. 10.1101/gad.11.23.3206 9389652 PMC316752

[B3] Alspaugh J. A. Perfect J. R. Heitman J. (1998). Signal transduction pathways regulating differentiation and pathogenicity of *Cryptococcus neoformans*. *Fungal Genet. Biol.* 25 1–14. 10.1006/fgbi.1998.1079 9806801

[B4] Alvarado-Ramirez E. Torres-Rodriguez J. M. Sellart M. Vidotto V. (2008). Laccase activity in *Cryptococcus gattii* strains isolated from goats. *Rev. Iberoam. Micol.* 25 150–153. 18785783 10.1016/s1130-1406(08)70035-4

[B5] Berman J. (2006). Morphogenesis and cell cycle progression in *Candida albicans*. *Curr. Opin. Microbiol.* 9 595–601. 10.1016/j.mib.2006.10.007 17055773 PMC3552184

[B6] Carreira A. Ferreira L. M. Loureiro V. (2001). Production of brown tyrosine pigments by the yeast *Yarrowia lipolytica*. *J. Appl. Microbiol.* 90 372–379. 10.1046/j.1365-2672.2001.01256.x 11298232

[B7] Chaskes S. Tyndall R. L. (1975). Pigment production by *Cryptococcus neoformans* from para- and ortho-Diphenols: effect of the nitrogen source. *J. Clin. Microbiol.* 1 509–514. 1100669 10.1128/jcm.1.6.509-514.1975 PMC275171

[B8] Chaskes S. Tyndall R. L. (1978). Pigment production by *Cryptococcus neoformans* and other *Cryptococcus species* from aminophenols and diaminobenzenes. *J. Clin. Microbiol.* 7 146–152. 344335 10.1128/jcm.7.2.146-152.1978 PMC274883

[B9] D’Souza C. A. Alspaugh J. A. Yue C. Harashima T. Cox G. M. Perfect J. R. (2001). Cyclic AMP-dependent protein kinase controls virulence of the fungal pathogen *Cryptococcus neoformans*. *Mol. Cell. Biol.* 21 3179–3191. 10.1128/MCB.21.9.3179-3191.2001 11287622 PMC86952

[B10] Eisenman H. C. Casadevall A. (2012). Synthesis and assembly of fungal melanin. *Appl. Microbiol. Biotechnol.* 93 931–940. 10.1007/s00253-011-3777-2 22173481 PMC4318813

[B11] Eisenman H. C. Mues M. Weber S. E. Frases S. Chaskes S. Gerfen G. (2007). *Cryptococcus neoformans* laccase catalyses melanin synthesis from both D- and L-DOPA. *Microbiology* 153 3954–3962. 10.1099/mic.0.2007/011049-0 18048910

[B12] Eisman B. Alonso-Monge R. Roman E. Arana D. Nombela C. Pla J. (2006). The Cek1 and Hog1 mitogen-activated protein kinases play complementary roles in cell wall biogenesis and chlamydospore formation in the fungal pathogen *Candida albicans*. *Eukaryot. Cell* 5 347–358. 10.1128/EC.5.2.347-358.2006 16467475 PMC1405885

[B13] Emery H. S. Shelburne C. P. Bowman J. P. Fallon P. G. Schulz C. A. Jacobson E. S. (1994). Genetic study of oxygen resistance and melanization in *Cryptococcus neoformans*. *Infect. Immun.* 62 5694–5697. 7960156 10.1128/iai.62.12.5694-5697.1994 PMC303323

[B14] Erickson T. Liu L. Gueyikian A. Zhu X. Gibbons J. Williamson P. R. (2001). Multiple virulence factors of *Cryptococcus neoformans* are dependent on VPH1. *Mol. Microbiol.* 42 1121–1131. 10.1046/j.1365-2958.2001.02712.x 11737651

[B15] Garcia-Rivera J. Eisenman H. C. Nosanchuk J. D. Aisen P. Zaragoza O. Moadel T. (2005). Comparative analysis of *Cryptococcus neoformans* acid-resistant particles generated from pigmented cells grown in different laccase substrates. *Fungal Genet. Biol.* 42 989–998. 10.1016/j.fgb.2005.09.003 16289955

[B16] Garcia-Rodas R. Cordero R. J. Trevijano-Contador N. Janbon G. Moyrand F. Casadevall A. (2014). Capsule growth in *Cryptococcus neoformans* is coordinated with cell cycle progression. *MBio* 5:e0094514 10.1128/mBio.00945-14 PMC4056547 24939886

[B17] Huerta-Cepas J. Bueno A. Dopazo J. Gabaldon T. (2008). PhylomeDB: a database for genome-wide collections of gene phylogenies. *Nucleic Acids Res.* 36 D491–D496. 10.1093/nar/gkm899 17962297 PMC2238872

[B18] Huerta-Cepas J. Capella-Gutierrez S. Pryszcz L. P. Denisov I. Kormes D. Marcet-Houben M. (2011). PhylomeDB v3.0: an expanding repository of genome-wide collections of trees, alignments and phylogeny-based orthology and paralogy predictions. *Nucleic Acids Res.* 39 D556–D560. 10.1093/nar/gkq1109 21075798 PMC3013701

[B19] Igual J. C. Johnson A. L. Johnston L. H. (1996). Coordinated regulation of gene expression by the cell cycle transcription factor Swi4 and the protein kinase C MAP kinase pathway for yeast cell integrity. *EMBO J.* 15 5001–5013. 8890173 PMC452238

[B20] Jacobson E. S. (2000). Pathogenic roles for fungal melanins. *Clin. Microbiol. Rev.* 13 708–717. 10.1128/CMR.13.4.708-717.2000 11023965 PMC88958

[B21] Jacobson E. S. Tinnell S. B. (1993). Antioxidant function of fungal melanin. *J. Bacteriol.* 175 7102–7104. 8226653 10.1128/jb.175.21.7102-7104.1993 PMC206840

[B22] Liu O. W. Chun C. D. Chow E. D. Chen C. Madhani H. D. Noble S. M. (2008). Systematic genetic analysis of virulence in the human fungal pathogen *Cryptococcus neoformans*. *Cell* 135 174–188. 10.1016/j.cell.2008.07.046 18854164 PMC2628477

[B23] Mauch R. M. Cunha Vde O. Dias A. L. (2013). The copper interference with the melanogenesis of *Cryptococcus neoformans*. *Rev. Inst. Med. Trop. Sao Paulo* 55 117–120. 10.1590/S0036-46652013000200009 23563765

[B24] Navarro-Garcia F. Eisman B. Fiuza S. M. Nombela C. Pla J. (2005). The MAP kinase Mkc1p is activated under different stress conditions in *Candida albicans*. *Microbiology* 151 2737–2749. 10.1099/mic.0.28038-0 16079350

[B25] Navarro-Garcia F. Sanchez M. Pla J. Nombela C. (1995). Functional characterization of the MKC1 gene of *Candida albicans*, which encodes a mitogen-activated protein kinase homolog related to cell integrity. *Mol. Cell. Biol.* 15 2197–2206. 7891715 10.1128/mcb.15.4.2197 PMC230448

[B26] Nosanchuk J. D. Casadevall A. (2003). The contribution of melanin to microbial pathogenesis. *Cell Microbiol.* 5 203–223. 10.1046/j.1462-5814.2003.00268.x 12675679

[B27] Nosanchuk J. D. Ovalle R. Casadevall A. (2001). Glyphosate inhibits melanization of *Cryptococcus neoformans* and prolongs survival of mice after systemic infection. *J. Infect. Dis.* 183 1093–1099. 10.1086/319272 11237835

[B28] Noverr M. C. Williamson P. R. Fajardo R. S. Huffnagle G. B. (2004). CNLAC1 is required for extrapulmonary dissemination of *Cryptococcus neoformans* but not pulmonary persistence. *Infect. Immun.* 72 1693–1699. 10.1128/IAI.72.3.1693-1699.2004 14977977 PMC356011

[B29] Nurudeen T. A. Ahearn D. G. (1979). Regulation of melanin production by *Cryptococcus neoformans*. *J. Clin. Microbiol.* 10 724–729. 44517 10.1128/jcm.10.5.724-729.1979 PMC273255

[B30] Perfect J. R. Lang S. D. Durack D. T. (1980). Chronic cryptococcal meningitis: a new experimental model in rabbits. *Am. J. Pathol.* 101 177–194. 7004196 PMC1903580

[B31] Plonka P. M. Grabacka M. (2006). Melanin synthesis in microorganisms–biotechnological and medical aspects. *Acta Biochim. Pol.* 53 429–443. 16951740

[B32] Pukkila-Worley R. Gerrald Q. D. Kraus P. R. Boily M. J. Davis M. J. Giles S. S. (2005). Transcriptional network of multiple capsule and melanin genes governed by the *Cryptococcus neoformans* cyclic AMP cascade. *Eukaryot. Cell* 4 190–201. 10.1128/EC.4.1.190-201.2005 15643074 PMC544166

[B33] Rodrigues M. L. Alvarez M. Fonseca F. L. Casadevall A. (2008a). Binding of the wheat germ lectin to *Cryptococcus neoformans* suggests an association of chitinlike structures with yeast budding and capsular glucuronoxylomannan. *Eukaryot. Cell* 7 602–609. 10.1128/EC.00307-07 18039942 PMC2292635

[B34] Rodrigues M. L. Nakayasu E. S. Oliveira D. L. Nimrichter L. Nosanchuk J. D. Almeida I. C. (2008b). Extracellular vesicles produced by *Cryptococcus neoformans* contain protein components associated with virulence. *Eukaryot. Cell* 7 58–67. 10.1128/EC.00370 18039940 PMC2224146

[B35] Roman E. Cottier F. Ernst J. F. Pla J. (2009). Msb2 signaling mucin controls activation of Cek1 mitogen-activated protein kinase in *Candida albicans*. *Eukaryot. Cell* 8 1235–1249. 10.1128/EC.00081 19542310 PMC2725568

[B36] Rosa L. H. Almeida Vieira Mde L. Santiago I. F. Rosa C. A. (2010). Endophytic fungi community associated with the dicotyledonous plant *Colobanthus quitensis* (Kunth) Bartl. (Caryophyllaceae) in Antarctica. *FEMS Microbiol. Ecol.* 73 178–189. 10.1111/j.1574-6941.2010.00872.x 20455944

[B37] Rosas A. L. Casadevall A. (1997). Melanization affects susceptibility of *Cryptococcus neoformans* to heat and cold. *FEMS Microbiol. Lett.* 153 265–272. 10.1016/S0378-1097(97)00239-5 9271852

[B38] Salas S. D. Bennett J. E. Kwon-Chung K. J. Perfect J. R. Williamson P. R. (1996). Effect of the laccase gene CNLAC1, on virulence of *Cryptococcus neoformans*. *J. Exp. Med.* 184 377–386. 10.1084/jem.184.2.377 8760791 PMC2192698

[B39] Takeo K. Tanaka R. Miyaji M. Nishimura K. (1995). Unbudded G2 as well as G1 arrest in the stationary phase of the basidiomycetous yeast *Cryptococcus neoformans*. *FEMS Microbiol. Lett.* 129 231–235. 7607405 10.1111/j.1574-6968.1995.tb07585.x

[B40] Tangen K. L. Jung W. H. Sham A. P. Lian T. Kronstad J. W. (2007). The iron- and cAMP-regulated gene SIT1 influences ferrioxamine B utilization, melanization and cell wall structure in *Cryptococcus neoformans*. *Microbiology* 153 29–41. 10.1099/mic.0.2006/000927-0 17185532

[B41] Vidotto V. Defina N. Pugliese A. Aoki S. Nakamura K. Takeo K. (2002). Effect of different K+ concentrations on *Cryptococcus neoformans* phenoloxidase activity. *Mycopathologia* 156 171–176. 10.1023/A:1023376324422 12749580

[B42] Virtudazo E. V. Kawamoto S. Ohkusu M. Aoki S. Sipiczki M. Takeo K. (2010). The single Cdk1-G1 cyclin of *Cryptococcus neoformans* is not essential for cell cycle progression, but plays important roles in the proper commitment to DNA synthesis and bud emergence in this yeast. *FEMS Yeast Res.* 10 605–618. 10.1111/j.1567-1364.2010.00633.x 20528951

[B43] Virtudazo E. V. Suganami A. Tamura Y. Kawamoto S. (2011). Towards understanding cell cycle control in *Cryptococcus neoformans*: structure-function relationship of G1 and G1/S cyclins homologue CnCln1. *Biochem. Biophys. Res. Commun.* 416 217–221. 10.1016/j.bbrc.2011.11.040 22119191

[B44] Wakamatsu K. Ito S. (2002). Advanced chemical methods in melanin determination. *Pigment. Cell Res.* 15 174–183. 10.1034/j.1600-0749.2002.02017.x 12028581

[B45] Walton F. J. Idnurm A. Heitman J. (2005). Novel gene functions required for melanization of the human pathogen *Cryptococcus neoformans*. *Mol. Microbiol.* 57 1381–1396. 10.1111/j.1365-2958.2005.04779.x 16102007

[B46] Wang X. Rocheleau T. A. Fuchs J. F. Christensen B. M. (2006). Beta 1, 3-glucan recognition protein from the mosquito, Armigeres subalbatus, is involved in the recognition of distinct types of bacteria in innate immune responses. *Cell Microbiol.* 8 1581–1590. 10.1111/j.1462-5822.2006.00732.x 16984413

[B47] Wang Y. Aisen P. Casadevall A. (1995). *Cryptococcus neoformans* melanin and virulence: mechanism of action. *Infect. Immun.* 63 3131–3136. 7622240 10.1128/iai.63.8.3131-3136.1995 PMC173427

[B48] Wang Y. Casadevall A. (1994a). Decreased susceptibility of melanized *Cryptococcus neoformans* to UV light. *Appl. Environ. Microbiol.* 60 3864–3866. 7986054 10.1128/aem.60.10.3864-3866.1994 PMC201897

[B49] Wang Y. Casadevall A. (1994b). Susceptibility of melanized and nonmelanized *Cryptococcus neoformans* to nitrogen- and oxygen-derived oxidants. *Infect. Immun.* 62 3004–3007. 8005689 10.1128/iai.62.7.3004-3007.1994 PMC302912

[B50] Waterman S. R. Hacham M. Panepinto J. Hu G. Shin S. Williamson P. R. (2007). Cell wall targeting of laccase of *Cryptococcus neoformans* during infection of mice. *Infect. Immun.* 75 714–722. 10.1128/IAI.01351 17101662 PMC1828480

[B51] White L. P. (1958). Melanin: a naturally occurring cation exchange material. *Nature* 182 1427–1428. 10.1038/1821427a0 13600352

[B52] Wollschlaeger C. Trevijano-Contador N. Wang X. Legrand M. Zaragoza O. Heitman J. (2014). Distinct and redundant roles of exonucleases in *Cryptococcus neoformans*: implications for virulence and mating. *Fungal. Genet. Biol.* 73 20–28. 10.1016/j.fgb.2014.09.007 25267175 PMC4382001

[B53] Zaragoza O. Casadevall A. (2004). Experimental modulation of capsule size in *Cryptococcus neoformans*. *Biol. Proced. Online* 6 10–15. 10.1251/bpo68 15103395 PMC389900

[B54] Zhu X. Gibbons J. Garcia-Rivera J. Casadevall A. Williamson P. R. (2001). Laccase of *Cryptococcus neoformans* is a cell wall-associated virulence factor. *Infect. Immun.* 69 5589–5596. 10.1128/IAI.69.9.5589-5596.2001 11500433 PMC98673

[B55] Zhu X. Gibbons J. Zhang S. Williamson P. R. (2003). Copper-mediated reversal of defective laccase in a Deltavph1 avirulent mutant of *Cryptococcus neoformans*. *Mol. Microbiol.* 47 1007–1014. 10.1046/j.1365-2958.2003.03340.x 12581355

